# A Comparison of Magnesium Levels in Obese Versus Normal-Weight Children

**DOI:** 10.7759/cureus.44053

**Published:** 2023-08-24

**Authors:** Basil A Alzahrani, Ziyad A Badri, Jamal A Aljuhani, Rayan M Alshamrani, Mohamed E Ahmed, Mesbah Jari Alshumrani

**Affiliations:** 1 King Saud Bin Abdulaziz University for Health Sciences, College of Medicine, King Abdullah International Medical Research Center, Jeddah, SAU; 2 Biostatistics, King Abdullah International Medical Research Center, Jeddah, SAU

**Keywords:** children, electrolyte, magnesium, obesity, pediatric

## Abstract

Introduction

One of the world's most pressing problems right now is childhood obesity. After potassium, magnesium (Mg) is the second most prevalent intracellular cation and the fourth most prevalent mineral in the human body. Numerous symptoms of magnesium insufficiency can include hypocalcemia, hypokalemia, as well as cardiac and neurological symptoms. Additionally, chronically low Mg levels have been associated with a number of chronic diseases such as diabetes, hypertension, coronary heart disease, and osteoporosis.

Objectives

This study aimed to compare the magnesium (Mg) level between normal-weight and obese children in a tertiary center in Saudi Arabia over the past seven years and evaluate the vitamin D and phosphorus between the two groups as a secondary objective.

Methods

This is a single-center, case-control study conducted on patients followed up in our center from January 2016 to December 2022. All pediatric patients were between two and 14 years of age. They were divided into two groups: one with children whose body mass index (BMI) was over the 85th percentile and the other with children whose BMI was between the 3rd and 85th percentiles.

Results

Mean serum Mg levels showed no significant correlation between the obese group (0.82 mg/dl) and the normal-weight group (0.83 mg/dl). However, vitamin D and phosphorus demonstrate a significant difference between the two groups. The obese group revealed a vitamin D of 1.6±0.24 and phosphorus of 4.2±0.46. On the other hand, the normal group had a vitamin D of 44.0±28.2 and phosphorus of 1.5±0.26.

Conclusion

There was a negative correlation between Mg levels and weight in pediatric patients. However, a positive relationship was observed between the Mg intake and Mg levels. Moreover, sodium, phosphorus, and vitamin D levels showed significant differences.

## Introduction

Obesity is becoming increasingly common in children and adolescents worldwide [1]. Childhood obesity is one of the most concerning issues facing the globe today. The problem is widespread and affects many poor- and middle-income countries, particularly metropolitan areas [2]. Childhood obesity results in various obesity-related illnesses, and its prevalence is increasing at an alarming rate. Globally, more than 107 million children are thought to be obese worldwide, with high-income nations having an obesity incidence of above 20%[3].

Obesity is defined as the accumulation of excess body fat to such an extent that health may be impaired (World Health Organization). Physicians define obesity in their daily practice as a body mass index (BMI) (body weight in kilograms divided by the square of height in meters) greater than 30, although BMI is not an accurate reflection of adipose mass. Children's BMI charts accounting for age and sex are also available. Several forms of obesity can be identified depending on the location of adipose tissue depots. Typically, obesity is characterized by an increase in adipocyte size and quantity. The predominant factor contributing to the development of obesity over time is overeating, rather than a problem with baseline energy expenditure, as evidenced by in vivo studies conducted on children and adolescents [4].

Chronic low-level inflammation, controlled by metabolic cells in response to an abundance of food, is associated with obesity. Immunometabolic illness has been linked to this inflammatory condition, which is prevalent in organs such as the liver, brain, pancreas, and adipose tissue. The adipose tissue contains a large number of immune cells, and obesity-related stimulation of the inflammatory response alters both the number and activity of these cells. As a result, inflammation and the immune system are out of control. This has been suggested to be the main mechanism linking obesity to vascular and metabolic side effects. It also helps explain why the risk of cancer and infectious diseases has increased. Moreover, through signaling pathways, gut microorganisms affect host metabolism and have an impact on insulin resistance, fat deposition, and inflammation. Obesity is associated with significant microbiological alterations [5].

Obesity in children and adolescents is linked to a higher risk of dying young. Poor academic performance may be caused by a variety of reasons, including depression, type 2 diabetes (T2D), various endocrine illnesses, cardiovascular disease, and cognitive impairments. Obesity affects nearly all organ systems in children, as well as depression and cognitive disturbances that may contribute to poor school results. The detrimental effects obesity may have on social interaction in youngsters is a further worry. Bullying is widespread, and negative attitudes toward obese kids may raise their risk of developing eating disorders, feeling isolated from their peers, and engaging in less physical exercise [6].

Magnesium (Mg) is the second most common intracellular cation after potassium and the fourth most common mineral in the human body after calcium, sodium, and potassium. Moreover, it is necessary for fundamental activities, including energy production and nucleic acid synthesis, and serves as a cofactor in more than 300 enzyme systems [7]. Mg deficiency can cause numerous manifestations such as hypocalcemia, hypokalemia, and cardiac and neurological indications. Additionally, various chronic disorders, including diabetes, hypertension, coronary heart disease, and osteoporosis, have been linked to persistently low Mg levels [8]. Additionally, several studies have shown a link between low Mg levels and increased oxidative and inflammatory stress. Recent studies have included obese participants in investigations linking low Mg levels to chronic inflammatory stress. Poor Mg levels have been linked to several chronic diseases, including type 2 diabetes, atherosclerosis, cancer, and obesity, which affect over 35% of the adult population in the United States [9]. Several studies have linked obesity to low serum Mg levels [10,11]. However, the relation between magnesium level and weight is still unclear.

## Materials and methods

This retrospective chart review study was conducted on patients followed up at King Abdulaziz Medical City, Jeddah, between 2016 and 2022. This study was approved by the Institutional Review Board of the King Abdullah International Medical Research Center (NRJ22J/299/11). The primary objective was to compare Mg levels between pediatric groups categorized as obese and non-obese based on the WHO growth chart; from the 3rd to the 85th percentile is normal weight and above the 85th percentile is obese. The inclusion criteria were patients aged two to 14 years with good social status having a recorded Mg level and a BMI higher than the 85th percentile. We also included a control group, aged two to 14 years, with Mg levels and a BMI in the 3rd to 85th percentile. Exclusion criteria comprised patients with genetic or endocrine diseases, diabetes mellitus, gastroenteritis, kidney disease, cancer, or those using amphotericin and steroids.

The sample size was calculated using Epitool's sample size online calculator for case-control studies. Considering a margin of error of 5%, a confidence interval of 95%, and odds ratios from previous relevant studies, it was determined that each patient group should consist of 92 participants, with a total sample size of 184. We included all pediatric patients who met the inclusion criteria with available Mg-level laboratory results since 2016. An electronic medical record system was established at our institution. Patient records were divided among data collectors. After completion of the data collection process, the accuracy of the data was independently reviewed by another author and the corresponding author.

Data were entered into a Microsoft Excel spreadsheet (Microsoft Corporation, Redmond, WA), and analysis was conducted using John’s Macintosh Project (JMP) Pro 15.2.0 statistical software (SAS Institute Inc., Cary, NC). Missing data and outlier values were detected and treated prior to the statistical analysis. Descriptive categorical variables were presented as frequencies and percentages. Descriptive numerical variables were depicted as means and standard deviations. For inferential statistics, the chi-square test, one-way analysis of variance (ANOVA), and unpaired t-test were used, with statistical significance denoted by a p-value set at p < 0.05.

## Results

The present study enrolled a total of 204 children, with 103 children categorized as obese and 91 as normal weight (Table 1). Among the obese group, 64 (62.1%) were male while 39 (37.9%) were female. The median and interquartile range (IQR) for age, height, weight, and BMI within the obese group were 9 (8), 137.5 (31), 46.2 (33), and 23.4 (6.1), respectively. Conversely, in the normal-weight group, 47 (51.6%) were male and 64 (62.1%) were female. The median and IQR for age, height, weight, and BMI within this group were 11.0 (8), 143.5 (40), 40.0 (28), and 18.0 (5.0), respectively. As illustrated in Table 2, a comparison between the obese and normal weight groups was performed using various variables. It was observed that significant differences existed in some variables, including Mg level after one year (P=0.001), vitamin D (P=0.01), phosphorus (P=0.001), and sodium (P=0.001). The mean and standard deviation values for the normal weight group were as follows: high-density lipoprotein (HDL) (1.69±1.3), T4 (13.1± 1.8), Mg level after one year (0.83±0.06), vitamin D (44.0±28.2), phosphorus (1.5±0.26), and sodium (137.7±2.). In contrast, for the obese group, the mean and standard deviation values were: HDL (1.2±0.21), T4 (15.2±1.3), Mg level after one year (2.4±0.12), vitamin D (1.6±0.24), phosphorus (4.2±0.46), and sodium (40.9±19.9). Nevertheless, no significant differences were noted in some variables, including glycated hemoglobin (HbA1c), random glucose, cholesterol, triglyceride, thyroid stimulating hormone, T4, and Mg levels.

**Table 1:** Basic characteristics of participants ^1 ^Median (IQR)

Variable	Normal	Obese	
	n=91	n=103	
Gender			
Male	47 (51.6)	64 (62.1)	
Female	44 (48.4)	39 (37.9)	
Age^1^	11.0 (8.0)	9.0 (8.0)	
Weight^1^	40 (28.0)	46.2 (33.0)	
Height^1^	143.5 (40.0)	137.5 (31.0)	
BMI^1^	18.0 (5.0)	23.4 (6.1)	

**Table 2:** Parameters by group HgA1c: glycated hemoglobin; HDL: high-density lipoprotein; TSH: thyroid-stimulating hormone

Variable	Normal	Obese	P-value
	n=91	n=103	
HgA1c	5.2± 0.49	5.5± 0.24	0.129
Random	5.6± 1.3	5.4± 1.0	0.262
Cholesterol	4.1± 0.78	4.1± 0.62	0.741
HDL	1.69± 1.3	1.2± 0.21	0.037
Triglyceride	0.99± 0.73	1.1± 0.65	0.450
TSH	2.5± 1.6	2.1± 2.3	0.403
T4	13.1± 1.8	15.2 ±1.3	0.423
Mg level	0.83± 0.06	0.82± 0.08	0.480
Mg at one year	0.83± 0.06	2.4± 0.12	0.001
Vitamin D	44.0± 28.2	1.6± 0.24	0.001
Phosphorus	1.5 ±0.26	4.2± 0.46	0.001
Sodium	137.7± 2.3	40.9± 19.9	0.001

**Figure 1:**
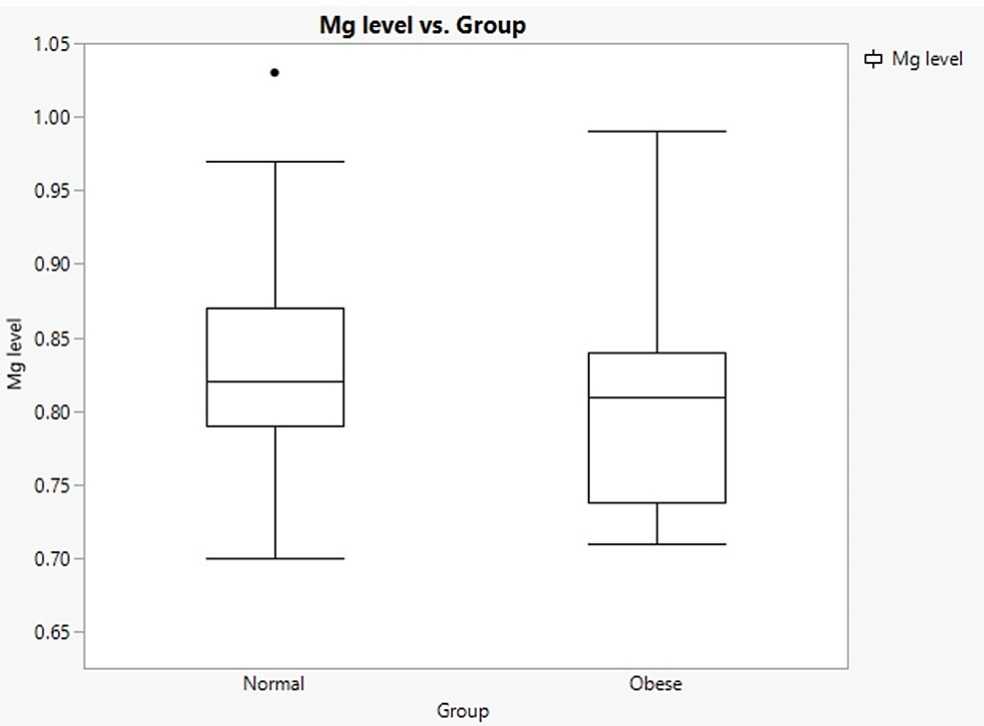
Mg level vs. group

**Figure 2:**
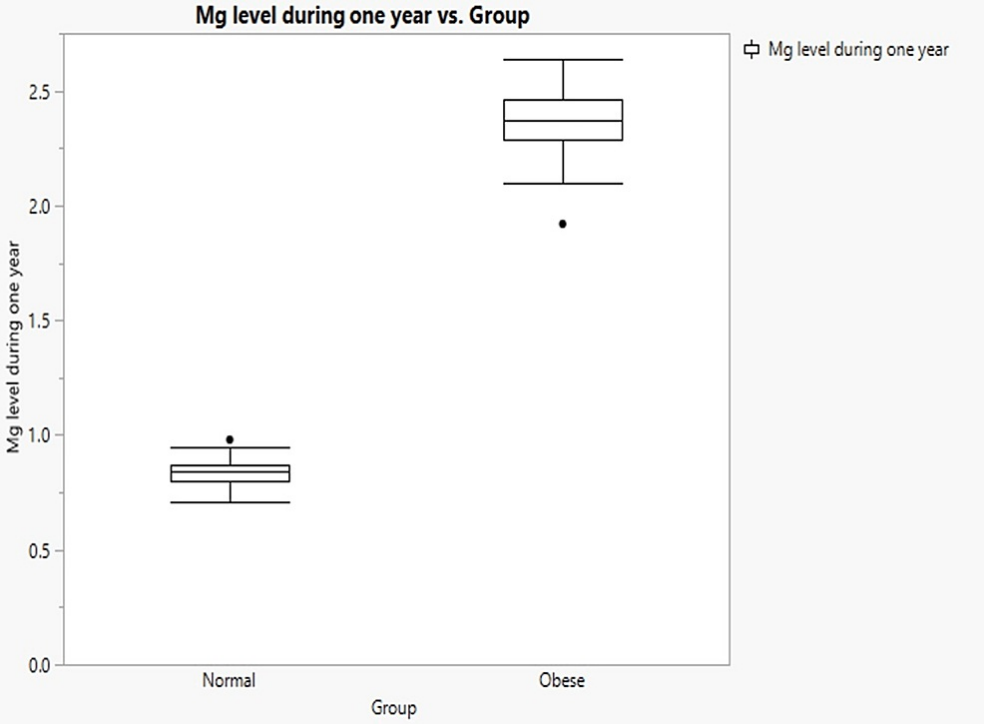
Mg level during one year vs. group

The results indicated that the mean serum Mg levels in the obese group and normal weight groups were 0.82 mg/dl and 0.83 mg/dl, respectively (Figure 1). The normal-weight group had higher Mg levels than the obese group. The P-value (0.48) was not statistically significant. Another significant result was the Mg level over a 12-month period, with a highly significant P-value of 0.001 (Figure 2). The Mg level was 2.4±0.12 for the obese group and 0.83±0.06 for the normal weight group. Consequently, the Mg level after one year was significantly higher in the obese group than in the normal-weight group.

## Discussion

Conditions such as genetic or endocrine diseases, diabetes mellitus, gastroenteritis, kidney disease, and cancer can affect the level of magnesium. This study included 103 obese children compared to 91 normal-weight children. The WHO growth chart was used to measure the percentile.

Various studies have been conducted on the relationship between Mg levels and multiple factors, including weight, age, sex, and race. In a study conducted by Huerta et al., the subjects were divided into four groups based on race [12]. Additionally, a cross-sectional study classified patients into two groups based on their diabetes status [13]. The subjects in another American study were divided into two age groups and two sex groups [14]. However, our findings are consistent with those of a study by Jose et al. who categorized participants into the normal-weight and overweight groups [15]. In that study, the mean BMI of the normal-weight group (14.8) was slightly lower than that of our group (18). Similarly, in another study, both groups had a mean BMI of 24 or higher, which could explain the variation in Mg levels [13]. Otherwise, the BMI was consistent with that in other studies. In all the previous studies, including ours, no statistical differences were observed among the groups in terms of age. Moreover, males were predominant in terms of sex distribution in all groups.

In our study, the Mg levels in the control and obese groups were not significantly different. This was comparable to Jie Wei’s cross-sectional study, which included 827 patients with a BMI greater than or equal to 25 kg, and revealed a negative correlation of 0.84 mmol/L. However, they found a positive correlation between Mg intake and Mg level, which was also evident in our results, ranging from 0.82 to 2.4 mmol/L [13]. Yet, a study done by Bertinato et al. showed mean serum Mg levels in overweight and obese children to be lower than those in normal-weight children. In this study, they categorized BMI groups, as follows: normal weight (BMI <23), overweight (BMI ≥23 -<27.5), and obese (BMI ≥27.5). Compared with normal (0.820.00 mmol/L, n=154) or overweight (0.820.01 mmol/L, n=112) females, obese females had lower serum Mg levels (0.780.01 mmol/L, P<0.05). Males who were normal weight (0.840.01 mmol/L, n=64), overweight (0.840.00 mmol/L, n=120), or obese (0.820.01 mmol/L, n=64) did not differ significantly from each other (P=0.05). When compared to females of normal weight (7.8%, n=12) or overweight (8.9%, n=10), obese females were more likely to have hypomagnesemia (28.9%, n=22; P<0.05). Males in the normal (3.1%, n=2), overweight (3.3%, n=4), and obese (4.7%, n=3) groups had hypomagnesemia (P<0.05). They explained that obese females had lower serum Mg levels than normal-weight or overweight women. Additionally, a higher proportion of obese females had hypomagnesemia. In males, serum Mg levels did not vary by BMI category, indicating that obesity is more detrimental to Mg levels in women [12]. This could explain our non-significant finding, as our study enrolled 64 males and 39 females.

Regarding vitamin D, our study showed a significant difference between normal and obese children (44.0± 28.2 vs. 1.6± 0.24, respectively). Research by Radulović et al. provided evidence in support of this. The mean vitamin D level was 53.23 in the obese group while in the normal group, it was 78.59 (P<0.001) [16].

Limitations

Since this is a retrospective study, it had all the drawbacks and restrictions that come with retrospective studies. Since all patients were included in this single-center trial, it is impossible to extrapolate the findings to a larger population. Our sample was reduced since some files were difficult to incorporate and contained incomplete data. Furthermore, other electrolytes should be evaluated in the next studies.

## Conclusions

In conclusion, our study revealed a negative correlation between Mg levels and weight in pediatric patients. However, a positive relationship was observed between Mg intake and Mg levels. Moreover, significant differences were observed in sodium, phosphorus, and vitamin D levels. Therefore, more research needs to be conducted on the correlation between weight and electrolytes, as well as the role of Mg intake in management strategies.
